# Evaluation of aortic stiffness with an oscillometric arteriograph device in patients with hypertension and ascending aortic aneurysm

**DOI:** 10.1590/1806-9282.20250481

**Published:** 2026-01-09

**Authors:** Abdulkadir Arpa, Mehmet Özbek, Tuncay Güzel, Nihat Polat

**Affiliations:** 1 Bismil State Hospital, Department of Cardiology – Diyarbakır, Turkey.; 2 Dicle University, Faculty of Medicine, Department of Cardiology – Diyarbakır, Turkey.; 3 Health Science University, Gazi Yaşargil Training and Research Hospital, Department of Cardiology – Diyarbakır, Turkey.

**Keywords:** Aortic stiffness, Hypertension, Pulse wave velocity, Aortic aneurysm, Echocardiography

## Abstract

**OBJECTIVE:**

The aim of this study was to compare central blood pressure, central pulse pressure, and parameters like augmentation index and pulse wave velocity, which are used to evaluate arterial stiffness using an oscillometric method called arteriography, among hypertensive patients with and without concomitant ascending aortic aneurysms.

**METHODS:**

This research was conducted as a single-center and prospective study. A total of 83 patients were included in this study, including 44 consecutive patients with hypertension diagnosed and an ascending aortic diameter of 40 mm or more detected by 2D transthoracic echocardiography, and 39 controls with hypertension diagnosed and a normal ascending aortic diameter.

**RESULTS:**

E/e’ ratio was found to be significantly higher in the aneurysm group (9.05±2.24 vs. 7.75±1.94, p<0.05). The difference between peripheral and central systolic blood pressures was found to be significantly lower in the aneurysm group compared to the control group (9.3±4.5 vs. 11.8±4.1, p<0.05, respectively). Among the examined arterial stiffness parameters, augmentation index values were significantly higher in the aneurysm group (27±8.7 vs. 22.7±9.6, p<0.05), while pulse wave velocity did not show a statistically significant difference between the groups (8.85±1.85 vs. 8.59±1.19, p>0.05, respectively).

**CONCLUSION:**

Our study suggests that a potential relationship between the development of ascending aortic aneurysm and elevated aortic stiffness in hypertensive patients.

## INTRODUCTION

Hypertension (HT) is a prevalent cardiovascular disease that ranks among the leading preventable causes of death worldwide. HT, commonly observed in society, causes damage in the heart, kidneys, brain, eyes, and vascular tissues. One such consequence is an aortic aneurysm. Its prevalence increases with age and is accelerated in the presence of HT^1^. HT leads to intimal loss of elastic fibers, extracellular matrix degradation, and smooth muscle necrosis, resulting in the stiffening and enlargement of the aortic wall. Consequently, the combination of advanced age and HT poses a significant risk for thoracic aortic aneurysms^2,3^.

Aortic stiffness is a parameter that reflects the elasticity and distensibility of the aortic wall. While this stiffness naturally increases with advancing age, certain cardiovascular risk factors can accelerate this process^4,5^. Arterial stiffness increases with HT, diabetes mellitus (DM), smoking, atherosclerosis, chronic kidney disease (CKD), and aging^5^. Research exploring the relationship between arterial stiffness and HT has suggested a cyclic interaction, implying that while HT might contribute to arterial stiffness development^6^.

Various methods are available for assessing arterial stiffness. Invasive measurements relying on catheters are challenging and expensive, prompting the development of non-invasive alternatives. Non-invasive methods such as arterial tonometry and, more recently, oscillometric arteriography devices are used to measure the augmentation index (Aix) and pulse wave velocity (PWV), both crucial parameters in determining arterial stiffness^7^.

In our study, we aimed to compare central blood pressure, central pulse pressure, and parameters like Aix and PWV, which are used to evaluate arterial stiffness using an oscillometric method called arteriography, among hypertensive patients with and without concomitant ascending aortic (AA) aneurysms.

## METHODS

### Study design

This research was conducted as a single-center and prospective study at the Department of Cardiology, Faculty of Medicine, Dicle University, between the dates of 15.02.2017 and 15.05.2017, following the approval from the Ethics Committee (approval number and date: 44/267, February 13, 2017). All patients gave written informed consent.

### Study population

A total of 83 patients were included in this study, including 44 consecutive patients with HT diagnosed and an AA diameter of 40 mm or more detected by 2D transthoracic echocardiography, and 39 controls with HT diagnosed and a normal AA diameter.

Individuals with bicuspid aortic valve, moderate to severe valve regurgitation or stenosis, prosthetic valves, left ventricular ejection fraction <50%, DM, coronary artery disease (CAD), under 18 years of age, known peripheral vascular disease, chronic liver or kidney failure, acute infection, genetic diseases, and patients with poor medication adherence were excluded from the study.

### Definitions

HT was defined according to the JNC 8 and ESC 2013 guidelines as systolic blood pressure (SBP) ≥140 mmHg and/or diastolic blood pressure (DBP) ≥90 mmHg, or the use of antihypertensive medication^8^. Twelve-lead electrocardiograms were obtained for individuals in both patient and control groups.

### Echocardiographic evaluation

Echocardiographic images of the enrolled patients were acquired in the left lateral decubitus position during expiration using a 1.7/3.4 MHz transducer (Vivid 6, General Electric, Horten, Norway), and they were assessed based on the guidelines of the American Society of Echocardiography (ASE) for left ventricular assessment^9^. In order to minimize potential measurement errors and inter-observer variability, all echocardiographic evaluations were conducted by an experienced cardiologist, and the final values were derived by averaging measurements obtained over multiple cardiac cycles.

### Arterial stiffness measurement

The Mobil-O-Graph device has been extensively compared with tonometric devices and invasive blood pressure measurements across diverse populations, and its accuracy is generally well accepted^10,11^. We employed a reliable, user-independent, oscillometric method using a single-cuff arterial graph device (Mobil-O-Graph PWA, I.E.M. GmbH, Germany) in our study. The device consisted of a cuff resembling those used for blood pressure measurement, sensors for pressure detection, and a computer for data processing. After entering each individual's birth date, height, and weight into the device's program, the cuff belonging to the device was placed on the upper arm of the individual in a sitting position, appropriate for the individual's arm circumference. The cuff was positioned at heart level, and the device automatically took three consecutive measurements at 30-s intervals. The arterial graph device completed these measurements in approximately 20–30 s and automatically calculated numerical data about peripheral blood pressures, central aortic pressures, Aix, and PWV.

Signals obtained through the oscillometric method were transferred to a computer via infrared wireless communication.

### Statistical analysis

We analyzed our data using SPSS for Windows version 18.0. Normal distribution of variables was assessed using visual and analytical methods. Descriptive statistics were expressed as mean±standard deviation for normally distributed data and as percentages for nominal data. Chi-square test was used to compare two or more group proportions. Comparison of means between two groups was performed using the independent samples t-test when parametric test assumptions were met, and the Mann-Whitney U test when they were not. Pearson's correlation analysis was used. Results in the study were evaluated at a significance level of p<0.05 with a 95%CI.

## RESULTS

Demographic, laboratory, and echocardiographic findings of the groups are given in Table 1. In the group of patients with aortic aneurysms, statistically significant higher values were observed in terms of left ventricular end-diastolic dimension (LVEDD) (48.1±3.71 vs. 45.7±3.14, p: 0.002), left ventricular end-systolic dimension (LVESD) (32.9±3.87 vs. 30.7±3.02, p: 0.004) parasternal long axis left atrium (PSLAX LA) diameter (48.7±4.93 vs. 46.4±4.42, p: 0.020), apical four-chamber left atrium (AP4) minör (38.6±4.23 vs. 36.7±3.47, p: 0.026) and major axis dimensions (48.7±4.93 vs. 46.4±4.42, p: 0.023).

**Table 1:** Demographic, laboratory, and echocardiographic findings of the groups.

Variables	Aneurysm group (n=44)	Control group (n=39)	p-value
Age (year)	59±11	58±10	0.765
Gender (male) n (%)	27 (61)	20 (51)	0.355
Hypertension duration (years)	7±6	6±5	0.194
Hyperlipidemia, n (%)	6 (14)	5 (13)	0.913
Cigarettes, n (%)	9 (21)	7 (18)	0.773
Body mass index (kg/m^2^)	29.4±5.1	31.3±4.5	0.085
Hemoglobin (g/dL)	14.1±1.9	14.5±1.8	0.344
HDL cholesterol (mg/dL)	47.3±12.4	45.9±10.8	0.585
LDL cholesterol (mg/dL)	106.1±36.4	118.4±35	0.121
Total cholesterol (mg/dL)	191.3±45	195.6±44.5	0.665
Triglyceride (mg/dL)	190.2±96.2	161±72.7	0.120
IVS (mm)	12.8±1.57	12.7±1.4	0.860
LVEDD (mm)	48.1±3.71	45.7±3.14	**0.002**
LVESD (mm)	32.9±3.87	30.7±3.02	**0.004**
PSLAX LA diameter (mm)	38.8±4.27	36.7±3.43	**0.020**
AP4 LA short diameter (mm)	38.6±4.23	36.7±3.47	**0.026**
AP4 LA long diameter (mm)	48.7±4.93	46.4±4.42	**0.023**
Ascending aorta (mm)	43.2±2.82	32.5±2.45	**<0.001**
LVEF (%)	60.2±1.05	60.1±0.80	0.634
E wave (m/sn)	0.70±0.15	0.69±0.12	0.914
A wave (m/sn)	0.85±0.19	0.82±0.15	0.484
E/A ratio	0.85±0.23	0.87±0.22	0.653
Lateral E speed (m/sn)	0.08±0.02	0.09±0.03	**0.021**
Lateral A speed (m/sn)	0.12±0.03	0.12±0.03	0.930
IVRT (msn)	79.8±18	82.0±14.4	0.535
E/e’ ratio	9.05±2.24	7.75±1.94	**0.006**
Betablocker, (%)	52.3	46.2	0.578
ACE-İ, ARB (%)	68.2	71.8	0.720
Calcium channel blocker, (%)	29.5	30.8	0.903
Diuretic, (%)	34.1	43.6	0.375

IVS: interventricular septum; LVEDD: left ventricular end-diastolic dimension; LVESD: left ventricular end-systolic dimension; PSLAX: parasternal long axis; AP4: anterior posterior 4; LA: left atrium; LVEF: left ventricular ejection fraction; IVRT: İsovolumetric relaxation time; HDL: high density lipoprotein; LDL: low density lipoprotein, ACE-İ: angiotensin converting enzyme-inhibitors, ARB: angiotensin II receptor blockers. Statistically significant values are denoted in bold.

Among the tissue Doppler parameters, the lateral a’ value did not show a statistically significant difference between the groups, while the lateral e’ value was significantly lower in the aneurysm group (0.08±0.02 vs. 0.09±0.03, p: 0.021). E/e’ ratio was found to be significantly higher in the aneurysm group (9.05±2.24 vs. 7.75±1.94, p: 0.006) (Table 1).

The blood pressure and arterial stiffness parameters of the groups are given in Table 2. The difference between peripheral and central SBPs was found to be significantly lower in the aneurysm group compared to the control group (9.3±4.5 vs. 11.8±4.1, p: 0.009). However, the difference between peripheral and central DBPs was not statistically significant between the groups (p>0.05).

**Table 2:** Blood pressures and arterial stiffness parameters of the groups.

Variables	Aneurysm group (n=44)	Control group (n=39)	p-value
Peripheral systole pressure (mmHg)	140±24	143±18	0.460
Peripheral diastole pressure (mmHg)	90±15	92±15	0.519
Mean arterial pressure (mmHg)	107±17	109±15	0.498
Heart rate, beats/min	78±12	80±14	0.557
Central systole pressure (mmHg)	130±21	131±17	0.847
Central diastole pressure (mmHg)	91±16	93±16	0.467
Central pulse pressure (mmHg)	40±12	38±10	0.451
Peripheral and central systolic pressure difference (mmHg)	9.3±4.5	11.8±4.1	**0.009**
Peripheral and central diastolic pressure difference (mmHg)	-1±1.5	-1.3±1.4	0.268
Augmentation index, @75 (%)	27±8.7	22.7±9.6	**0.038**
Pulse wave rate (m/s)	8.85±1.85	8.59±1.19	0.449

Statistically significant values are denoted in bold.

Among the examined arterial stiffness parameters, Aix values were significantly higher in the aneurysm group (27±8.7 vs. 22.7±9.6, p: 0.038), while PWV did not show a statistically significant difference between the groups (8.85±1.85 vs. 8.59±1.19, p>0.05) (Table 2).

Correlation analysis of demographic, echocardiographic, and blood pressure parameters with arterial stiffness parameters between the groups is given in Table 3. In the aneurysm group, PWV showed a positive significant correlation with age (r: 0.894, p<0.001), left atrium diameter (r: 0.401, p: 0.007), diastolic mitral A wave velocity (r: 0.474, p: 0.001), and peripheral and central systolic pressure differences (r: 0.483, p: 0.001). On the other hand, the E/A ratio showed a significant negative correlation (r: −0.549, p<0.001) (Table 3).

**Table 3:** Correlation analysis of demographic, echocardiographic, and blood pressure parameters with arterial stiffness parameters between groups.

Variables	Pulse wave velocity				Augmentation index			
	Aneurysm (+)		Aneurysm (-)		Aneurysm (+)		Aneurysm (-)	
	r	p	r	p	R	P	r	p
Age	0.894	<0.001	0.657	<0.001	0.312	0.039	0.085	0.606
LVEF	-0.245	0.109	-0.220	0.178	-0.063	0.683	-0.234	0.151
Left atrium diameter	0.401	0.007	-0.125	0.447	0.291	0.056	0.001	0.994
Ascending aortic diameter	0.172	0.263	0.216	0.187	0.131	0.398	0.095	0.566
A wave speed	0.474	0.001	0.258	0.113	0.191	0.215	0.267	0.100
E/A ratio	-0.549	<0.001	-0.157	0.341	-0.375	0.012	-0.335	0.037
PWV	1	1	1	1	0.373	0.013	0.352	0.028
Aix@75	0.373	0.013	0.352	0.028	1	1	1	1
Central pulse pressure	0.322	0.033	0.358	0.025	0.195	0.205	0.219	0.180
Peripheral and central systolic pressure difference	0.483	0.001	0.255	0.118	0.231	0.132	-0.088	0.596

LVEF: left ventricular ejection fraction; PWV: pulse wave velocity.

In the aneurysm group, there was a positive significant correlation between Aix and age (r: 0.312, p: 0.039), while a negative significant correlation was observed between Aix and E/A ratio (r: −0.375, p: 0.012).

## DISCUSSION

In our study, the most significant finding was the increased aortic stiffness in patients with coexisting aneurysms and HT compared to those without aneurysms. Age, independent of cardiovascular risk factors and blood pressure, is a fundamental factor determining the stiffness of large elastic arteries, especially beyond the age of 55 years^12^. This is because with advancing age, there is remodeling and deterioration of elastic components in the arterial wall. As a result, increased arterial stiffness is observed in both older men and women. In our study, we also observed a significant correlation between age and arterial stiffness markers such as PWV and Aix in both the aneurysm and control groups.

However, the development of arterial stiffness is not solely determined by age. Notably, the significant variations in arterial stiffness among different populations and patient groups of the same age indicate the influence of various factors on the development of arterial stiffness. In our study, since the patients did not have clinical conditions (DM, CKD, CAD) other than HT, the duration of HT was similar between the two groups, and there was no significant difference between other demographic characteristics. The results obtained suggest that aneurysm development in the HT patient group may be related to aortic stiffness.

Systemic arterial HT increases aortic stiffness and leads to left ventricular hypertrophy and diastolic dysfunction^13^. The E/E’ ratio of the left ventricle is one of the indicators of diastolic function and increases with the progression of diastolic dysfunction, correlating with left ventricular diastolic pressure^14^. Moreover, an increased E/E’ ratio has been associated with reduced aortic compliance^15^. In our study, the E/E’ ratio was found to be significantly higher in the aneurysm group, indicating increased diastolic dysfunction, potentially linked to elevated aortic stiffness. This is consistent with a study by Mottram et al. where aortic compliance was independently predictive of diastolic dysfunction^16^.

Palmieri et al. reported that increased arterial stiffness contributed to the enlargement of left ventricular and left atrial dimensions, the development of diastolic dysfunction, atrial fibrillation, and stroke^17^. In our study, we similarly found that the aneurysm group exhibited significantly higher Aix values and increased left atrial and left ventricular dimensions.

In addition, in the aneurysm group, PWV was significantly positively correlated with left atrial diameter and diastolic mitral A wave velocity, while it was negatively correlated with the E/A ratio. This supports the idea that aortic stiffness is associated with left ventricular diastolic dysfunction in patients with aortic aneurysms.

Aix represents the distribution of arterial wave reflections and is associated with aortic blood pressure^18^. In our study, we found significantly higher Aix values in the aneurysm group, suggesting increased aortic stiffness. The correlation between Aix, PWV, and age in our study suggests that Aix might be a useful parameter in assessing arterial stiffness in this patient group.

A study focusing on thoracic aortic aneurysms and dissections has found significantly higher Aix values in patients with aortic pathology compared to normal individuals^19^. This aligns with our study's findings of elevated Aix in the aneurysm group.

A study has shown that as age progresses, the flexibility of the arterial system decreases and the difference between peripheral and central blood pressure decreases^20^. In our study, where the average age of patients was similar, the difference between peripheral and central SBP was found to be significantly lower in the aneurysm group compared to the control group. This result supports the idea of increased arterial stiffness in the aneurysm group compared to the control group.

In conclusion, our study highlights the relationship between aortic aneurysms, arterial stiffness, and various cardiovascular parameters. The results suggest that coexisting HT and aneurysms contribute to increased aortic stiffness, which may impact overall cardiovascular health. The comprehensive analysis of age, arterial stiffness markers, and cardiovascular risk factors helps shed light on the complex interplay between these factors and their implications for the pathophysiology of aortic aneurysms.

## LIMITATIONS

Our study indeed demonstrates several limitations that should be acknowledged in the interpretation of our findings. One significant limitation is the relatively small sample size in our study. Larger sample sizes generally contribute to more statistically robust results and allow for a better representation of the population under investigation. Additionally, conducting a single-center study could introduce bias related to the specific characteristics of the patient population at that center. Multi-center studies can offer a broader perspective and enhance the generalizability of findings.

Comparing echocardiographic measurements of AA diameter to those obtained from computed tomography (CT) scans is another limitation. While echocardiography is a valuable tool, CT scans are generally considered more accurate for assessing aortic dimensions.

Another limitation is that some variables that may influence aortic stiffness and aneurysm progression, such as lifestyle factors (e.g., diet and exercise) and genetic predispositions, are not taken into account.

## CONCLUSION

It is clear from our study that patients with both HT and AA aneurysm exhibit increased arterial stiffness, and this is associated with diastolic dysfunction of the left ventricle. Furthermore, our study suggests that the use of the osilometric arteriograph device is a simple, cost-effective, and reliable method for assessing increased aortic stiffness in this patient group.

## Data Availability

The datasets generated and/or analyzed during the current study are available from the corresponding author upon reasonable request.
